# Maternal mortality in Kassala State - Eastern Sudan: community-based study using Reproductive age mortality survey (RAMOS)

**DOI:** 10.1186/1471-2393-11-102

**Published:** 2011-12-16

**Authors:** Abdalla A Mohammed, Mahgoub H Elnour, Eltayeb E Mohammed, Samah A Ahmed, Ahmed I Abdelfattah

**Affiliations:** 1 Department of Obstetrics and Gynecology, University of Kassala, P.O. Box 266, Kassala Sudan; 2 Kassala State Ministry of Health, 3111 Kassala, Sudan; 3 Department of Community Medicine, University of Kassala, P.O. Box 266, Kassala, Sudan; 4 Reproductive Health, Ministry of Health, Kassala State, 3111 Kassala, Sudan

## Abstract

**Background:**

The maternal mortality ratio in Sudan was estimated at 750/100,000 live births. Sudan was one of eleven countries that are responsible for 65% of global maternal deaths according to a recent World Health Organization (WHO) estimate. Maternal mortality in Kassala State was high in national demographic surveys. This study was conducted to investigate the causes and contributing factors of maternal deaths and to identify any discrepancies in rates and causes between different areas.

**Methods:**

A reproductive age mortality survey (RAMOS) was conducted to study maternal mortality in Kassala State. Deaths of women of reproductive age (WRA) in four purposively selected areas were identified by interviewing key informants in each village followed by verbal autopsy.

**Results:**

Over a three-year period, 168 maternal deaths were identified among 26,066 WRA. Verbal autopsies were conducted in 148 (88.1%) of these cases. Of these, 64 (43.2%) were due to pregnancy and childbirth complications. Maternal mortality rates and ratios were 80.6 per 100,000 WRA and 713.6 per 100,000 live births (LB), respectively. There was a wide discrepancy between urban and rural maternal mortality ratios (369 and 872\100,000 LB, respectively). Direct obstetric causes were responsible for 58.4% of deaths. Severe anemia (20.3%) and acute febrile illness (9.4%) were the major indirect causes of maternal death whereas obstetric hemorrhage (15.6%), obstructed labor (14.1%) and puerperal sepsis (10.9%) were the major obstetric causes.

Of the contributing factors, we found delay of referral in 73.4% of cases in spite of a high problem recognition rate (75%). 67.2% of deaths occurred at home, indicating under utilization of health facilities, and transportation problems were found in 54.7% of deaths.

There was a high illiteracy rate among the deceased and their husbands (62.5% and 48.4%, respectively).

**Conclusions:**

Maternal mortality rates and ratios were found to be high, with a wide variation between urban and rural populations. Direct causes of maternal death were similar to those in developing countries. To reduce this high maternal mortality rate we recommend improving provision of emergency obstetric care (Emoc) in all health facilities, expanding midwifery training and coverage especially in rural areas.

## Background

World Health Organization (WHO)s 10th revision of the International Statistical Classification of Diseases and Related Health Problems (ICD-10) defines maternal mortality as "the death of a woman while pregnant or within 42 days of termination of pregnancy irrespective of the duration and the site of the pregnancy, from any cause related to or aggravated by the pregnancy or its management but not from accidental or incidental causes" [1]. Every year, approximately 358,000 women die from complications of pregnancy and childbirth worldwide. Sub-Saharan Africa and South Asia accounted for 87% of global maternal deaths [2]. According to a recent WHO estimate, Sudan is one of 11 countries that are responsible for 65% of global maternal deaths, with a maternal mortality ratio of 750/100,000 live births [2]. Because there is no accurate vital registration system in Sudan, maternal mortality estimates in Sudan were based on indirect and direct sisterhood estimates as in the demographic surveys conducted in the past four decades [3-5] and WHO estimates [2,6-8].

Maternal mortality in Kassala State was estimated to be high in all of these surveys. In a national household health survey conducted in 2006, the maternal mortality ratio was found to be 1,414 per 100,000 live births [4]. The causes of maternal mortality was mainly derived from hospital data [9]; however, only 16.2% of deliveries occur in hospitals [4]. This study was conducted to investigate the causes of maternal deaths on a community basis. It also looked at the rates and contributing factors of maternal deaths and compare the maternal deaths between urban and rural areas in the State.

## Methods

We conducted a reproductive age mortality survey (RAMOS) in Kassala State from 2004 - 2006. The study consisted of a retrospective community-based survey with two phases of data collection: death identification and interviews of respondents from the deceased household using a standard verbal autopsy questionnaire [10].

At the time of the study, the population of Kassala State was 1,768,603, with 70% of people living in rural areas. Of these, 424,465 were women of reproductive age (WRA), and there were 49,521 annual expected live births. Reproductive health services were delivered through primary health care system that composed of primary health care units, health centers and rural hospitals. There were three tertiary hospitals. The majority of health centers and rural hospitals were poorly staffed, and cannot deliver emergency obstetric care (Emoc). Deliveries were conducted by certified village midwifes at home and the majority of deliveries in the rural areas were conducted by traditional birth attendants.

We selected four areas in the state that represented the urban\rural ratio, remoteness and population diversity. We used a methodology similar to that used by Bartlett in Afghanistan in her purposive study area selection [11]. The selected areas were considered the primary sampling unit (PSU) of the study. The population of these areas was 335,394 (19% of the total population). These areas consisted of 130 villages and neighborhoods and 21,450 households. Villages and neighborhoods constitute the secondary sampling units (SSU) in this study.

The sample size was chosen based on the expected number of maternal deaths. Based on the maternal mortality in Kassala State in previous studies, we expected 268 annual maternal deaths in the state. We sought to detect 5-8% of these deaths, or between 40 and 80 deaths, over the three years of the study, which is sufficient as recommended by World Health Organization (WHO) [12].

Simple randomization using random tables was used to select 32 villages and neighborhoods, which constituted our study sample. The population of the study sample was 105,105 (31.3% of the PSU). Table 1 summarizes the demographic data of the SSU & PSU.

**Table 1:** Denominator data of study area in Kassala State - East Sudan

Indicator	Value
*Kassala State*	

Total population	1,768,603

*Primary sampling unit*	

Total population	335,394

Urban	219,454 (65.4%)

Rural	115,940 (34.6%)

Number of villages and neighborhoods	130

Number of households	21,450

Women of reproductive age	80,494

Expected live births	9,391

*Secondary sampling unit*	

Total population	105,105

Number of villages and neighborhoods	32

Number of households	5,795

Women of reproductive age	26,066

Expected live births	2,943

In phase one of this study, deaths were identified by trained field investigators who interviewed key informants in the community and asked about deaths of women of reproductive age (WRA) who died during the period from January 1, 2004 to December 31, 2006. The key informants were persons known to have access to detailed information about the local community such as community leaders, health workers or primary school teachers. The key informants were asked by field investigators to recall women deaths in their surroundings. To minimize recall error, each key informant was asked to give a list of female deaths, from which a single list was developed. Field investigators collected data on a death identification form that included the location name, name of data collector, name and age of the deceased, name of household members who attended the last illness and death of the deceased, date of death and household address. They prepared lists from different independent key informants. Different lists were attained for each village, and a final list was constructed. The research team examined the collected forms and selected suitable respondents for interview. The respondents were adult members of the household of the deceased, close relatives or neighbors or a local health worker who attended the death.

Phase two consisted of verbal autopsies in which trained interviewers visited the households of the deceased and interviewed predetermined respondents using a standard verbal autopsy questionnaire [10]. A narrative history of the circumstances surrounding the death was recorded from the respondent, and structured questions were asked regarding personal information, socio-economic background, place of death, treatment received for illness before death and symptoms of the deceased. An informed verbal consent was received from the deceased first of kin and from a respondent.

Data on the cause of death were analyzed using SPSS version 12 in relation to socio-economic characteristics of the household, place of death, birth attendant and type of treatment received. Each author (A.A.M., M.H.E & S.A.M) independently assigned the cause of death by reading the collected data. Denominator data was obtained from Kassala State Expanded Program of Immunization (EPI) data as shown in Table 1.

The study protocol was approved by the Kassala State Ethical Committee.

## Results

During the period 2004-2006, a total of 168 women aged 15-45 years were identified as dead in phase one in the study area. One hundred forty eight (88.1%) households of these women were visited, and respondents were interviewed. The reproductive age mortality rate was calculated as 188/100,000 WRA. Sixty four cases were classified as maternal deaths, that represent the proportion of maternal deaths among female of reproductive age (PMDF) of (43.2%). Maternal mortality rate and ratio were found to be 80.6/100,000 WRA & 713.6/100,000 live births (LB). Maternal mortality ratio in rural and urban areas were 872 and 369/100,000 LB respectively. The majority of deaths occurred in the 25-29 age group (Table 2). The deceased age ranged from 16-43 years, with median age of 26.5 years and a standard deviation of 6.3 years.

**Table 2:** Age distribution of 64 maternal deaths in Kassala State (2004-2006)

Age group	n	%
< 20	16	25.0

20-24	5	07.8

25-29	22	34.4

30-34	10	15.6

35-39	8	12.5

40+	3	04.7

Total	64	100.0

Table 3 represents the causes of deaths. Twenty-six deaths (41.6%) were due to indirect obstetric causes of which anemia, acute febrile illness and jaundice were the major indirect causes. In 38 cases (59.4%), we found a direct obstetric cause of maternal death. Severe anemia is the major cause of indirect maternal death and accounts for 20.3% of cases. forty three cases (67.2%) died at home with small number (04.7%) died on their way to health facility. Forty five (70.3%) died in the postnatal period, 25% died undelivered and only three (04.7%) in early weeks of pregnancy from complications of abortion. Reproductive characteristics are shown in Table 4. Thirty-eight (59.4%) cases did not receive antenatal care. Nearly 70% of those who died in the postpartum period delivered at home, half delivered by non-skilled attendants, and a similar number of pregnancies resulted in live birth. Three cases delivered on their way to the hospital. Two of these cases were cases of obstetric hemorrhage. Vaginal delivery constituted 93.3% of deliveries. There were three postoperative deaths; two were due to sepsis following caesarean section for obstructed labor, and one was a case of a ruptured uterus.

**Table 3:** Cause of maternal death in 64 cases in Kassala State (2004-2006)

Cause	n	%
Direct obstetric causes		

Obstetric hemorrhage	10	15.6

Obstructed labor	9	14.1

Puerperal sepsis	7	10.9

HDP	5	07.8

Abortion	4	06.3

Miscellaneous	3	04.7

Indirect causes:		

Severe anemia	13	20.3

Acute febrile illness	6	09.4

Jaundice	4	06.3

Miscellaneous	3	04.7

Total	64	100.0

HDP, hypertensive disease with pregnancy

**Table 4:** reproductive characteristics & pregnancy outcome in 64 maternal deaths in Kassala State (2004-2006)

Characteristic	n	%
*Parity (n = 64)*		

Nullipara	16	25.0

Multipara	32	50.0

Grandmultipara	16	25.0

*Antenatal care (n = 64)*		

Yes	21	32.8

No	43	67.2

*Place of delivery (n = 45)*		

Home	31	68.9

Health facility	11	24.4

On the way	3	06.7

*Skill attendant at birth (n = 45)*		

Yes	22	48.9

No	23	51.1

Mode of delivery *(n = 45)*		

Vaginal	43	93.3

Caesarean section	3	06.7

*Pregnancy outcome (n = 45)*		

Live birth	23	51.1

Still birth	18	40.0

Neonatal death	2	08.9

Of the 45 deceased who gave birth before they died, 31 (68.9%) delivered at home, and three (6.7%) delivered on their way to the hospital. Of these, 22 (49.9%) attended by unskilled person.

All of the deceased were married women. Table 5 shows the demographic characteristics of these deaths. The majority of cases were of low socio-economic class. Fifty-three (82.8%) deaths occurred in the rural areas. Sixty (93.8%) cases were not engaged in income generating activity. Of the deceased, 90.6% and 82.2% of their husbands were either illiterate or had non-formal education i.e that they are able to write and read without formal schooling.

**Table 5:** Demographic characteristics in 64 maternal deaths in Kassala State (2004-2005)

Characteristic	n = (64)	%
*Residence category*		

Urban	11	17.2

Rural	53	82.8

*Deceased education*		

Illiterate	40	62.5

Non-formal	18	28.1

Elementary	2	03.1

Secondary	3	04.6

Higher	1	01.6

*Husband education*		

Illiterate	31	48.4

Non-formal	28	43.8

Elementary	1	01.6

Secondary	3	04.6

Higher	3	04.6

*Husband occupation*		

Farmer	23	36.0

Worker	15	23.4

Sheppard	13	20.3

Other	13	20.3

**The first phase delay: **(delay in seeking medical care): In forty eight cases (75.0%) the respondents recognized a medical problem at the same time there was a delay in seeking medical care in 73.4%. They mentioned that there was no service available in the nearby health facilities, including the hospitals and they cannot afford to go to the tertiary hospital. The decision to seek medical care usually made by the husband or male household leader, who is usually away during the day; therefore, the woman has to wait until his returns at the evening to seek medical care. In one case, a woman with postpartum hemorrhage due to retained placenta bled for seven hours while waiting for her husband to return home. She died while being taken to the hospital.

**The second phase delay: **(Delay in reaching health facility): there were few paved roads in the State, beside some seasonal rivers that delay reaching to health facilities. Rural hospital were not equipped with ambulances. We found transportation problems in 54.7% of cases.

**The third phase delay: **forty four (68.8%) of them reported delay in receiving medical care at the level of primary health facility. Respondents mentioned unavailability of emergency drugs, absence of health workers and late referral to higher level.

## Discussion

To our knowledge, this is the first community-based study using a reproductive age mortality survey (RAMOS) in any part of Sudan on the state level. The maternal mortality ratio was found to be 714\100,000 live births. The proportion of maternal deaths among female deaths (PMDF) in our study was 43.3%, which is high compared to the reported national estimate of 19.4% [2]. This high rate of maternal mortality reflects poor maternity services. We found a wide discrepancy between urban and rural areas (369 & 872/100,000 LB, respectively). Even in urban areas, sectoral differences in maternal mortality were reported [9] in which slum dwellers and internally displaced people camps around cities had a high maternal mortality ratio compared to their neighboring inhabitants. More than 75% of deaths occurred during childbirth and postpartum, which is consistent with the pattern of causes in Sub-Saharan Africa. For example, in Eritrea, Ghebrehiwot [13] found that 16% of maternal deaths occurred during pregnancy, 48% occurred during childbirth, and 36% occurred postpartum.

Indirect causes account for 20 to 25% of maternal deaths and are attributable to illnesses aggravated by pregnancy [6] such as anemia, malaria and acquired immune deficiency syndrome (HIV/AIDS). In our study, indirect causes constituted more than 40% of deaths, with severe anemia as the major cause. Anemia is highly prevalent in Africa, with up to three-fifths of pregnant women having some degree of anemia and approximately one-third classified as having severe anemia [14-17]. Anemia is common in Eastern Sudan due to malaria, chronic illnesses and poverty [18] and is the main risk factor for stillbirth in maternity hospitals in Kassala [19]. Women, especially those in rural areas in Kassala State, enter pregnancy in a state of nutritional deficit and therefore are unprepared to cope with the extra physiological demands of pregnancy. Mason et al. found anemia in 45% of pregnant women and 49% of children under age 5 in developing countries [20]. Anemia is also an underlying risk factor for 18% of maternal and 24% of perinatal mortality [21,22].

The direct obstetrical causes of death in our study, obstetric hemorrhage, obstructed labor, sepsis and hypertensive disease with pregnancy, were similar to those of developing countries [23]. Abortion accounts for a small percentage of the deaths in this study. This is primarily because the respondents have difficulty recognizing the early menstrual history of the deceased. Also induced abortion for unwanted and out of wedlock pregnancy were usually not revealed due to influence of culture and abortion law.

The most common cause of maternal death was bleeding, which can kill even a healthy woman within two hours if unattended. Anemia in pregnant women reduces a woman's ability to survive bleeding during and after childbirth. In this study, hemorrhage is the second most common cause of death. These women likely faced bleeding in a state of anemia.

The "Three Delays" model [24], which includes delays in the decision to seek care, delays in reaching care, and delays in receiving care at the facility, were evident in our study. Although problem recognition was high, we found that the delay in seeking care was also high. In this study, 67.2% of deaths occurred at home. These patients were either unable or did not want to reach the health facility.

During the period of the study, the state was affected by war, and roads were closed by night. These factors likely contributed to transportation problems, which were found in 54.7% of the cases.

Delay in receiving medical care at health facilities, primarily due to lack of emergency obstetric care (Emoc) in almost all the health facilities in the rural settings, was found in 68.8% of cases.

Domestic delivery is common in Sudan, which was estimated at 76.5% and 82% nationally and in Kassala State, respectively [4]. In rural settings in Kassala State, almost all deliveries occur at home, and patients seek medical care if life-threatening complications arise. More than half of those who died after delivery were delivered by non-skilled birth attendants. Sudan Household Health Survey (SHHS) showed that 49.2% of births in the two years prior to the survey were delivered by qualified health personnel. The majority of the qualified health personnel are village midwives, who are on the margin of the health system in Sudan. They have no fixed jobs in the health system and depend on the incentives given by parturient mothers. Inability of health facilities to deliver effective Emoc led the community to lose trust in these hospitals, and these health facilities became a distinct cause of delay. Other interactive factors such as poverty, illiteracy and transportation problems were contributed to deaths in this study.

This high percentage of maternal deaths could be effectively reduced by improving the availability and use of Emoc in all health facilities. There is a need to expand midwifery coverage by availability of a certified midwife in every village. We recommend expansion of midwifery training by opening midwifery schools in remote areas. Establishing maternity waiting home near the tertiary hospitals will enable patients from rural areas with obstetric complications to stay in the town and avoid long cost hospitalization. To overcome transportation problems, rural hospital needs to be equipped with ambulances. Furthermore, improving non-health sector factors such as poverty, female education and infrastructure is important to reduce maternal mortality in the state.

## Conclusion

Maternal mortality rates and ratios were found to be high, with a wide variation between urban and rural populations. The proportion of maternal deaths among female deaths (PMDF) was higher than expected. Indirect causes of maternal death were high, and anemia was the major cause of indirect maternal death. Direct causes of maternal death were similar to those in developing countries. To reduce this high maternal mortality rate we recommend improving provision of Emoc in all health facilities, expanding midwifery training and coverage especially in rural areas. To overcome transportation problems, rural hospital needs to be equipped with ambulances. Furthermore, improving non-health sector factors such as poverty, female education and infrastructure is important to reduce maternal mortality in the state."

## Competing interests

The authors declare that they have no competing interests.

## Authors' contributions

AAM generated the idea of the research, performed the literature review and contributed to writing and editing of the manuscript. MHE field supervisor, contributed to analysis and writing of manuscript. SAA act as field supervisor and financial director and contributed to analysis and writing of the manuscript. EEM & AIA contributed with other authors in assignment of cause of maternal death from verbal autopsy data. All author read and approved the final manuscript..

## Pre-publication history

The pre-publication history for this paper can be accessed here:

http://www.biomedcentral.com/1471-2393/11/102/prepub
